# Dual-Threshold-Based Microstate Analysis on Characterizing Temporal Dynamics of Affective Process and Emotion Recognition From EEG Signals

**DOI:** 10.3389/fnins.2021.689791

**Published:** 2021-07-14

**Authors:** Jing Chen, Haifeng Li, Lin Ma, Hongjian Bo, Frank Soong, Yaohui Shi

**Affiliations:** ^1^School of Computer Science and Technology, Faculty of Computing, Harbin Institute of Technology, Harbin, China; ^2^Shenzhen Academy of Aerospace Technology, Shenzhen, China; ^3^Speech Group, Microsoft Research Asia, Beijing, China; ^4^Heilongjiang Provincial Hospital, Harbin, China

**Keywords:** EEG, dual-threshold-based AAHC, microstate characteristics, auditory emotion process, emotion recognition

## Abstract

Recently, emotion classification from electroencephalogram (EEG) data has attracted much attention. As EEG is an unsteady and rapidly changing voltage signal, the features extracted from EEG usually change dramatically, whereas emotion states change gradually. Most existing feature extraction approaches do not consider these differences between EEG and emotion. Microstate analysis could capture important spatio-temporal properties of EEG signals. At the same time, it could reduce the fast-changing EEG signals to a sequence of prototypical topographical maps. While microstate analysis has been widely used to study brain function, few studies have used this method to analyze how brain responds to emotional auditory stimuli. In this study, the authors proposed a novel feature extraction method based on EEG microstates for emotion recognition. Determining the optimal number of microstates automatically is a challenge for applying microstate analysis to emotion. This research proposed dual-threshold-based atomize and agglomerate hierarchical clustering (DTAAHC) to determine the optimal number of microstate classes automatically. By using the proposed method to model the temporal dynamics of auditory emotion process, we extracted microstate characteristics as novel temporospatial features to improve the performance of emotion recognition from EEG signals. We evaluated the proposed method on two datasets. For public music-evoked EEG Dataset for Emotion Analysis using Physiological signals, the microstate analysis identified 10 microstates which together explained around 86% of the data in global field power peaks. The accuracy of emotion recognition achieved 75.8% in valence and 77.1% in arousal using microstate sequence characteristics as features. Compared to previous studies, the proposed method outperformed the current feature sets. For the speech-evoked EEG dataset, the microstate analysis identified nine microstates which together explained around 85% of the data. The accuracy of emotion recognition achieved 74.2% in valence and 72.3% in arousal using microstate sequence characteristics as features. The experimental results indicated that microstate characteristics can effectively improve the performance of emotion recognition from EEG signals.

## Introduction

To make a human–machine interaction more natural, emotion recognition should play an important role. Interest in emotion recognition from different modalities (e.g., face, speech, body posture, and physiological responses) has risen in the past decades. Physiological signals could measure the changes in physiological responses to emotional stimulus. They have advantages on eliminating social masking or factitious emotion expressions to obtain a better understanding of underlying emotions (Jang et al., 2015). Among the various types of physiological signals, an electroencephalogram (EEG) shows a direct measure of the electrical activity of the brain. It has been used in cognitive neuroscience to investigate the regulation and processing of emotion (Dennis and Solomon, 2010; Thiruchselvam et al., 2011). With the rapid development of dry EEG electrode techniques, EEG-based emotion recognition has received increasing applications in different fields such as affective brain–computer interaction (Atkinson and Campos, 2016; Chen et al., 2021), healthcare (Hossain and Muhammad, 2019), emotional companionship, and e-learning (Ali et al., 2016).

Early work on emotion recognition from EEG goes back as far as 1997 (Musha et al., 1997). In the past several years, various signal processing methods have been proposed to improve the EEG-based emotion recognition. Previous studies (Jenke et al., 2014; Alarcao and Fonseca, 2017) provided a comprehensive overview of the existing works in emotion recognition based on EEG signals. Feature extraction is a critically significant step in EEG-based emotion recognition framework. Basically, features from EEG can be distinguished in time domain, frequency domain, and time–frequency domain. The time domain features aim to identify and detect the temporal information in the brain activity. Frantzidis et al. (2010) used amplitude and latency of event-related potentials (ERPs) as features for EEG-based emotion classification. However, it is difficult to detect ERPs related to emotions since the onset is usually unknown. Other features, such as Hjorth features (Mehmood and Lee, 2015), fractal dimension (Sourina and Liu, 2011; Liu and Sourina, 2013), and higher-order crossings (Petrantonakis and Hadjileontiadis, 2009) have been used to characterize EEG time series. The frequency–domain feature aims to capture the relative amplitude and phase information of specific oscillation frequency. The most popular frequency–domain features are band power (Rozgić et al., 2013) and high-order spectra (Hosseini et al., 2010). These features could be extracted from different frequency bands, e.g., delta (1–3 Hz), theta (4–7 Hz), alpha (8–13 Hz), beta (14–30 Hz), and gamma (31–49 Hz). With this kind of method, it is not possible to determine when a particular frequency occurs. The time–frequency domain features bring up the temporal information by considering the dynamical changes of spectrum. The most commonly used time–frequency analyses for feature extraction were short-time Fourier transform (Lin et al., 2010), wavelet transform (Mohammadi et al., 2017), and Hilbert–Huang transform (Zong and Chetouani, 2009).

However, some limitations still exist on traditional feature sets. As EEG is an unsteady and rapidly changing voltage signal, the feature extracted from EEG usually changes dramatically, whereas emotion states change gradually (Wang et al., 2014). This leads to bigger differences among EEG features, even with the same emotion state in adjacent time. Most existing feature extraction approaches do not consider these differences between EEG and emotion. In this study, the authors proposed a feature extraction method based on EEG microstates for emotion recognition. Microstate analysis treats multichannel EEG as a series of momentary quasi-stable scalp electric potential topographies (Pascual-Marqui et al., 1995). These quasi-stable potential topographies are referred to as microstates, so brain electrical activity could be modeled as being composed of a time sequence of non-overlapping microstates. Microstate sequences could capture the important spatio-temporal properties of an EEG signal. At the same time, it can reduce the fast-changing EEG signals to a sequence of prototypical topographical maps. Characterizing the dynamics of brain neuronal activity through EEG microstate patterns could provide novel information for improving EEG-based emotion recognition.

Microstate analysis has been used to study the resting state of the human brain based on the topography of the EEG signals (Van de Ville et al., 2010; Khanna et al., 2015; Michel and Koenig, 2018). The greater part of the literature acknowledges four standard microstate maps on healthy subjects at rest. In addition, the characteristics of microstate sequences have been proven to offer a potential biomarker for some diseases, such as mood and anxiety disorders (Al Zoubi et al., 2019), autism spectrum disorder (D’Croz-Baron et al., 2019), and schizophrenia (Soni et al., 2018, 2019; da Cruz et al., 2020; Kim et al., 2021). Baradits et al. (2020) created a specified feature set to represent microstate characteristics. These features were used to classify patients with schizophrenia and healthy controls.

While microstate analysis has been widely used to study brain function, few studies have used this method to analyze how the brain responds to emotional auditory stimuli. There are some challenges when applying microstate analysis to emotion process. Considering the complex emotion process, how to determine the optimal number of microstates automatically is a subject worthy of study. The modified K-means and K-medoids had been used to determine the microstate classes in many studies (Von Wegner et al., 2018). However, these methods need pre-set K cluster centers, and the clusters are sensitive to the initialization. Emotional response is a complex cognitive process so that it is difficult to predict the number of microstate classes subjectively. Atomize and agglomerate hierarchical clustering (AAHC) algorithm is specifically proposed for the microstate analysis of EEG (Murray et al., 2008). It is a hierarchical clustering that can offer more optional clustering results. The method initializes with a large number of clusters and then reduces the number of clusters by one during each iteration step. It stops when only one single final cluster is obtained, but the best partition from numerous clustering results is subjectively determined.

**FIGURE 1:**
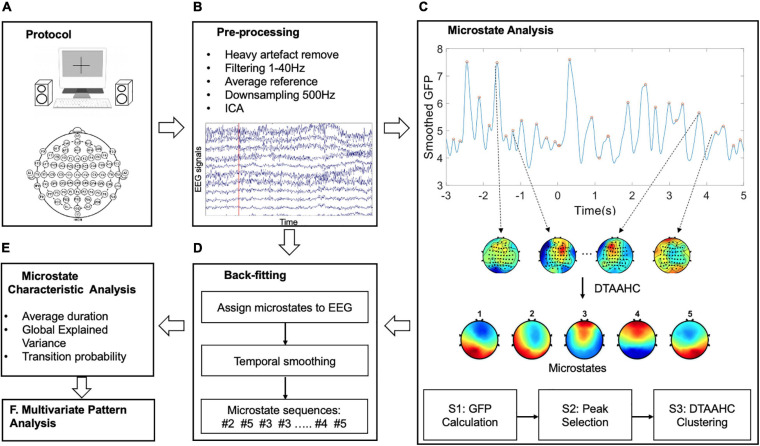
The schema of the methodology. The main six steps are: **(A)** The auditory emotional experimental design. **(B)** The pre-processing for EEG signals. **(C)** The proposed microstate analysis to identify the microstates. **(D)** Back-fitting to obtain the microstate sequences. **(E)** Microstate characteristics extraction as features. **(F)** Multivariate pattern analysis for emotion recognition.

To overcome this limitation, this study proposes a dual threshold-based atomize and agglomerate hierarchical clustering (DTAAHC) which can determine the optimal number of microstate classes automatically. For microstate analysis, microstates are expected to be distinct and could explain the original EEG topographies as much as possible. Therefore, two optimization criteria are used to estimate the quality of the candidate microstates during iterations. Compared with AAHC, in addition to global explained variance (GEV) contribution, the proposed algorithm also considers the microstate topographic similarity. Global map dissimilarity (GMD) is used to measure the topographic differences of candidate microstates. In addition, the iteration stops when the criterion GEV reaches the threshold. Although we made a minor alteration to the AAHC algorithm, the new method could identify the optimal microstate classes automatically and reduce the computational cost. By using the proposed method to model the temporal dynamics of the auditory emotion process, we extract microstate characteristics as novel temporospatial features for improving the performance of emotion recognition from EEG signals. The schema of the present study is shown in Figure 1.

## Materials and Methods

This section provides details of the experimental tasks and datasets used in this study. In addition, we describe the proposed DTAAHC and the temporal parameters of microstate sequences for emotion recognition.

### Datasets

Speech, music, and ambient sound events carry emotional information in human communication. In the present study, we focused on the emotional response induced by speech and music. Two independent datasets were available for analysis.

#### Dataset 1: Speech-Evoked Emotion Cognitive Experiment

##### Participants

Nineteen healthy participants (8 females and 11 males) with normal hearing participated in the experiment. The mean age of the 19 subjects was 22.4 (SD = 5.4; range, 18–27) years. All subjects were self-reported right-handers. All subjects had no personal history of neurological or psychiatric illness. The subjects were undergraduate and graduate students at Harbin Institute of Technology. The participants must exhibit enough proficiency in English. The ethics committee of Heilongjiang Provincial Hospital accepted the study. The concept was explained to the subjects, and written informed consent was obtained.

##### Stimuli selection

There are two unique models for signifying emotions: the categorical model and the dimensional model. In the former, emotions are recognized with the help of words denoting emotions or class tags. In the dimensional model, the representation is based on a set of quantitative measures using multidimensional scaling. One of the classical and widely used categorical models is six basic emotion classes, namely, anger, disgust, fear, joy, sadness, and surprise (Ekman et al., 1987). Various dimensional models have also been proposed (Schlosberg, 1954; Russell and Mehrabian, 1977; Russell, 1980). In this work, we use the valence–arousal scale of Russell (1980), which is widely used in research on affect, to quantitatively describe emotions. In this scale, each emotional state can be placed on a two-dimensional plane with arousal and valence as the horizontal and vertical axes, respectively. In the present research, we first selected stimuli by categorical model. After selection, we rated the valence–arousal scales for each stimulus online using Self-Assessment Manikin (SAM).

Considering the six basic emotions, we collected 20 pairs of audio clips for each emotion category. Each pair of clips was the same slice of a film in two languages (original English version vs. Chinese-dubbed version).

The stimuli used in the experiment were selected in three steps. First, we selected the raw films by watching a range of films for 1 month. The principles considered in the raw film selection are listed below: (A) The films display relatively strong emotions; (B) The films should have an original English version and a Chinese-dubbed version; and (C) The Chinese-dubbed version matches the original version to the greatest extent. We finally selected 40 films as raw sources. Second, we need to select emotional clips from the films. This step is carried out manually. The selection requirements are as follows: (A) Each segment should contain the speech of only one speaker; (B) Each segment expresses a single desired target emotion; (C) Each segment lasts for 5 s and contains at least a complete utterance; and (D) The background sound should not be too obvious. We finally selected 158 pairs of clips. We extracted soundtracks from these film clips. Third, all the audio clips were manually rechecked to guarantee the quality of emotional expression by 10 subjects. Some clips with ambiguous emotions were removed. We finally selected 20 pairs of clips for each emotion category which maximize the strength of elicited emotions. The list of the film clips is shown in Supplementary Table 1.

To obtain reliable emotional labels of these clips, we utilized Amazon’s Mechanical Turk service to collect data from native English-speaking and native Chinese (Mandarin)-speaking subjects. We initially started with a target goal of 40 repetitions per clip. The subjects were allowed to classify as many of the 240 possible audio clips as they wish. There was no expectation for a single subject to complete all 240 audio exemplars. In the event that a subject completes only a portion of the 240 audio clips, we will continue to solicit additional subjects until we have achieved the required number of responses.

**FIGURE 2:**
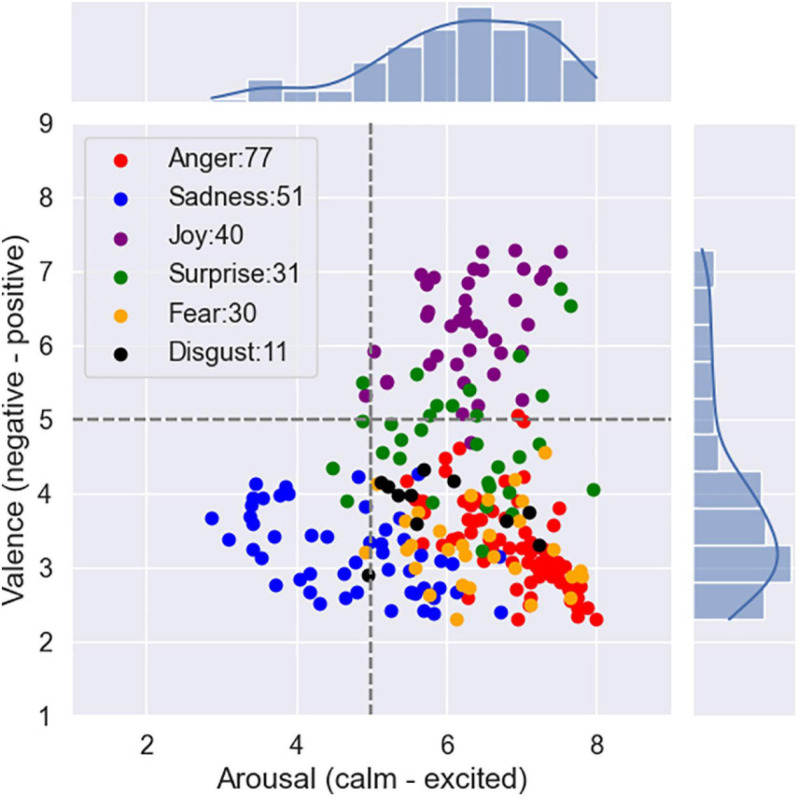
The distribution of ratings on arousal–valence plane.

We presented subjects with selected audio clips and asked them to rate the emotional content of what they just heard and how they arrived at that decision. Discrete affective label and dimensional emotional annotation (arousal–valence) with 1–9 scales related to a single audio clip were obtained. Figure 2 shows the mean locations of the stimuli on the arousal–valence plane.

##### Experimental protocol

Before the experiment, the subjects were given a set of instructions to help them understand the experiment protocol. When the instructions were clear, the participants were led into the experiment room with sensors placed on their heads. After that, an experimenter explained the meaning of the different scales of SAM. The SAM is a non-verbal pictorial assessment technique that directly measures the valence, arousal, and dominance associated with the affective reaction of a person to a wide variety of stimuli. The arousal dimension ranges from a relaxed, sleepy figure to an excited, wide-eyed figure. The valence dimension ranges from a frowning, unhappy figure to a smiling, happy figure. The dominance–submissiveness scale represents the controlling and dominant vs. controlled or submissive one feels: a prominent figure indicates maximum control in the situation. The participants could perform three practice trials to familiarize themselves with the experiment.

**FIGURE 3:**
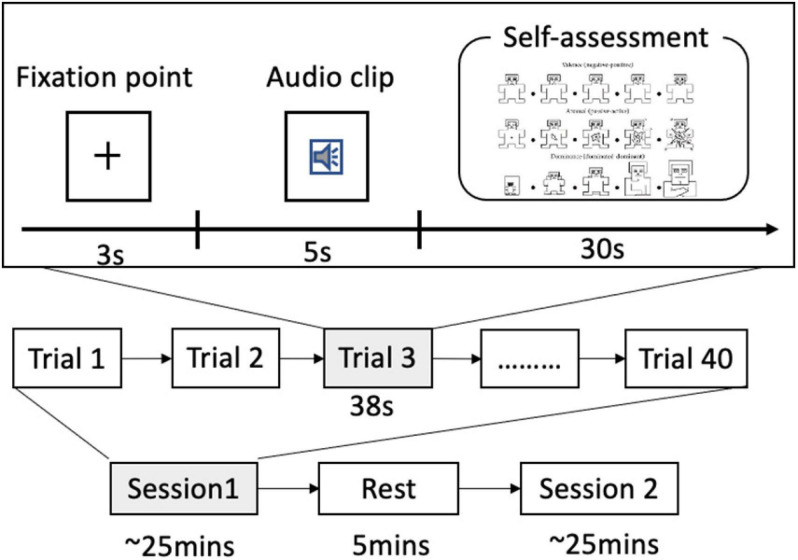
The process of speech-evoked emotion cognitive experiment.

The subjects were instructed to keep their eyes open for the entire duration of the experiment. The process of our experiment is depicted in Figure 3. In this experiment, each subject performed two sessions of around 25 min each. They can have a 5-min break after one session is finished. Each session consisted of 40 trials.

Audio clips inducing different emotional states were presented in random order. Each trial consists of the following steps:

(a) a 3-s baseline recorded, during which the subjects were instructed to watch a fixation cross presented on a computer monitor,
(b) a 5-s audio clip played, during which the subjects were instructed to listen attentively and watch a central visual fixation, and
(c) a 30-s self-assessment for arousal, valence, and dominance, during which the subjects used a computer keyboard to rate the SAM on a scale of 1–9.

The experiment was programmed using Psychophysics Toolbox of Matlab. Table 1 summarizes the number of trials for high/low valence and arousal and the average rating for the four conditions.

**TABLE 1:** Database summary.

	Valence		Arousal	
Condition	High	Low	High	Low
Number of trials	790	583	815	558
Rating	5.9 ± 0.8	3.3 ± 0.5	6.4 ± 0.7	3.7 ± 0.3

##### EEG acquisition

The EEG signals were continuously recorded using a 64-channel EEG system (64-channel Quik-Cap and Neuroscan Synamp2 Amplifier). The cap had 64 electrodes and two integrated bipolar which led for vertical and horizontal electrooculography (EOG). During recording, two EOGs and two mastoid electrodes (M1 and M2) were not placed. Each electrode impedance should be less than 10 kΩ. The sampling rate was 1,000 Hz. The electrodes were placed over the scalp according to the international 10–20 system.

##### EEG pre-processing

The EEG signal pre-processing was performed to reduce unwanted noise and artifacts that compromise the quality of the signal. First, four signals from two EOGs and two mastoid electrodes were removed. Sixty-two remaining signals were used for the processing and analysis of the next step. Then, the EEG signals were average-referenced, down-sampled to 500 Hz, and filtered with 1–35 Hz to obtain the desired frequency range and remove the electrical line noise. After that, the eye blinks and muscular artifacts were excluded using independent component analysis (ICA). For each group, each participant, and each trial, EEG signal from 3-s baseline before the audio clip was removed to correct stimulus-unrelated variations. The pre-processing was performed using EEGLAB of Matlab.

#### Dataset 2: Music-Evoked Emotion Cognitive Experiment

Music is a powerful method for emotional communication and can evoke genuine basic emotions in the listener (Daly et al., 2015). Physiological measurements can be used to identify personal emotional responses to music. A popular public database, Dataset for Emotion Analysis using Physiological signals (DEAP), has been widely used to analyze affective states (Koelstra et al., 2011). DEAP is a multimodal dataset, including EEG, MEG, galvanic skin resistance, electrooculography, blood volume pressure, skin temperature, and respiration pattern. A total of 32 subjects participated in the data collection, and 40 carefully pre-selected 1-min-long music videos were used as the stimulus to elicit emotions for each subject. Before each video is displayed, a 5-s baseline is recorded. Each participant was requested to finish a self-assessment for arousal, valence, and dominance on a scale of 1–9 after watching.

In this research, we used 32-channel EEG original signals for emotion recognition based on microstate analysis. The raw EEG data can be downloaded from http://www.eecs.qmul.ac.uk/mmv/datasets/deap/. During pre-processing, the EEG data was average-referenced, down-sampled to 128 Hz, and filtered with a 1–35-Hz cutoff, and eye artifacts were removed with ICA. The 5-s baseline before the stimuli was used to correct the data for stimulus-unrelated variations. There is a total of 1,280 trials for analysis.

### The Proposed Dual-Threshold-Based Microstate Analysis

The principles of microstate analysis are the quasi-stable periods of topographies, which is demonstrated in previous studies. More particularly, the changes of electric field configurations can be described by a limited number of microstate classes, which remain stable for around 80–120 ms before abruptly transitioning to another configuration. EEG microstates might represent and characterize the dynamic neuronal activity of conscious contents.

#### Global Field Power

Global field power (GFP) is calculated to find a series of dominant template topographies. GFP constitutes a single, reference-independent measure of response strength at a global level (Lehmann and Skrandies, 1980). GFP is simply the standard deviation of all electrodes at a given time. What GFP tells the researcher is, on average across the electrode montage, how strong is the potential being recorded. It is often used to measure the global brain response to an event or to characterize the rapid changes in brain activity.

For each subject, GFP was calculated for each sample time according to Eq. 1, where *N* denotes the number of electrodes, *u*_*i*_(*t*) is the measured voltage of a specific electrode at time *t*, and $\bar{u\left(t\right)}$ is the average voltage of the *N* electrodes at the respective sample time *t*.

(1) $$GFP\left(t\right)=\sqrt{\frac{\sum_{i=1}^{N}{\left(u_{i}\left(t\right)-\bar{u\left(t\right)}\right)}^{2}}{N}}$$

The local maxima of the GFP curve represent high global neuronal synchronization (Skrandies, 2007) and are considered with the highest signal-to-noise ratio. The topographies around these peaks remain stable and are submitted to the clustering algorithm. For each participant, the GFP of each trial is calculated. After smoothing the GFP with a Gaussian-weighted moving average of 50 time points, topographies at GFP peaks were collected and fed into a DTAAHC clustering algorithm to identify the microstates.

#### The Proposed Dual-Threshold-Based AAHC

AAHC is a bottom-up hierarchical clustering wherein the number of clusters is initially large and progressively diminishes. Classical agglomerative hierarchical clustering would eventually disintegrate the short-duration period of stable topography. These short-duration periods would be designated to other clusters even if they contribute a high GEV (Murray et al., 2008). In AAHC, clusters are given priority according to their GEV contributions. In this way, short-duration periods are conditionally maintained. Specifically, during each iteration, AAHC frees the cluster with the lowest GEV and then re-assigns these “free” maps to the surviving clusters by calculating spatial correlation. The iterations stop when only one single final cluster is obtained. An important next step is the choice of the number of desired output clusters. Unfortunately, there is no definitive solution. The more clusters one identifies, the higher the quality of the clustering but the lower the data reduction. Five criteria to decide on the amount of microstate clusters have been described by Poulsen et al. (2018). GEV is used to measure the percentage of data that can be explained by microstate classes. The cross-validation criterion is related to the residual noise. Dispersion (*W*) is a measure of the average distance between members of the same cluster. However, it is not a suitable measure of fitting for polarity-invariant methods such as modified K-means and AAHC. Krzanowski–Lai criterion and normalized Krzanowski–Lai criterion are based on dispersion (*W*).

Here we propose DTAAHC to determine the optimal number of microstate classes automatically during clustering. Compared with AAHC, in addition to GEV contribution, the proposed algorithm also considers the microstate topographic similarity. For microstate analysis, microstates are expected to be distinct and could explain the original EEG topographies as much as possible. Therefore, two optimization criteria are used to estimate the quality of the topographical maps of microstate classes during iterations. First, the cluster with the lowest GEV is freed and re-assigned to the surviving clusters. Second, the clusters are merged if the GMD between the candidate microstate classes is lower than 0.1. In addition, the iteration stops when the criterion GEV reaches the threshold. Although we made a minor alteration to the AAHC algorithm, the new method could identify the optimal microstate classes automatically and reduce the computational cost. The detailed introduction of this method is discussed below.

GMD is used to measure the topographic differences of microstate maps, independent of electric strength. It is defined as follows:

(2) $$\mathrm{GMD}=\sqrt{\frac{1}{N}\sum_{i=1}^{N}{\left(\frac{u_{i}-\bar{u}}{GFP_{u}}-\frac{v_{i}-\bar{v}}{GFP_{v}}\right)}^{2}}$$

where *u_i* and *v_i* are the voltages of two specified microstates, and$\bar{u}$ and $\bar{v}$ are the average voltages of the *N* electrodes. GMD ranges from 0 to 2, where 0 indicates topographic homogeneity and 2 indicates topographic inversion.

GEV measures the percentage of data that can be explained by microstate classes. It is frequently used to quantify how well the microstate classes describe the whole data. The higher GEV, the better. It is influenced by the dimensionality of the data. The total GEV is the sum of the GEV values over all microstate classes:

(3) $$GEV=\sum_{l}GEV_{l}$$

The GEV_*l*_ value for a specific microstate class with label *l* is:

(4) $${\text{GEV}}_{l}=\frac{\sum_{t}GFP_{t}^{2}\cdot C_{V_{t},M_{l}}^{2}\cdot \delta_{l,L_{t}}}{\sum_{t}GFP_{t}^{2}}$$

(5) $$\delta_{l,L_{t}}=\left\{ \begin{array}{c} 1\;ifl=L_{t}\\ 0\;ifl\neq L_{t} \end{array}\right.$$

(6) $$C_{V_{t},M_{l}}=\frac{\sum_{i}V_{ti}M_{li}}{\sqrt{\sum_{i}V_{ti}^{2}}\cdot \sqrt{\sum_{i}M_{li}^{2}}}$$

The spatial correlation *C*_*V_t,M_l*_between instantaneous EEG topography *V_t* and the candidate microstate class *M_l* can be calculated by Eq. 6, where *V*_*ti*_ is the voltage of *i*th electrode of instantaneous EEG at time *t* (local peak index), and *M*_*li*_ denotes the topography of the microstate class *l*.

In this study, DTAAHC is performed on the EEG topographies at local peaks of GFP. During initialization, each topography map is considered as a unique cluster. Upon subsequent iterations, the spatial correlation *C*_*V_t,M_l*_ between each instantaneous EEG topography *V_t* and the candidate microstate class *M_l* will be calculated by Eq. 6, merging the clusters which have maximum spatial correlation. The groups of the centroid of maps are defined as the candidate microstate class for that cluster. Then, two optimization criteria are applied. The GEV_*l*_ for a specific microstate class with label *l* is calculated by Eq. 4. The cluster with the lowest GEV is removed and re-assigned to the most similar cluster during each iteration step. The GMDs between the candidate microstate classes are calculated. The clusters are merged if the GMD is lower than the threshold. The iterations stop when the GEV is higher than the threshold. In the present work, the threshold of GEV is set to 85% (Lehmann et al., 2005; Michel and Koenig, 2018; D’Croz-Baron et al., 2019). The threshold of GMD is set to 0.1 (Murray et al., 2008). Table 2 shows the DTAAHC procedure.

**TABLE 2:** Pseudocode for dual-threshold-based atomize and agglomerate hierarchical clustering (DTAAHC).

**Algorithm:** DTAAHC

**Inputs:** set of *n* topographies *D*{*S*_1_,*S*_2_,*S*_3_,,*S*_*n*_};
the spatial correlation *C*;
Th_*GEV*_: threshold of global explained variance (GEV)
Th_*GMD*_: threshold of global map dissimilarity
**Procedure:**
1: **for** *i* = 1, 2, …, *n* **do**
2: *Cluster*_*i*_{*S*_*i*_}
3: **end for**
4: **repeat**
5: **for** *i* = 1, 2, …, *n* **do**
6: **for** *j* = 1, 2, …, *n* **do**
7: *C*_*Cluster_i, Cluster_j*_ = *C*(Cluster_*i*_,_Cluster*j*_)
8: **end for**
9: **end for**
10: merge two clusters ${\text{Cluster}}_{i^{*}}$ and ${\text{Cluster}}_{j^{*}}$, which have maximum spatial correlation max(*C*_Cluster_*i*_,*Cluster*_*j*__)
11: define the centroid (mathematical average) as the template map for that cluster
12: calculate the GEV_*l*_ between each template map and samples
13: the cluster with the min (GEV_*l*_) is atomized, and each sample in this cluster is independently re-assigned to the surviving cluster with the highest spatial correlation
14: calculate the GMD for each pair of template map
15: merge clusters if the GMD is lower than Th_*GMD*_
16: **until** sum (GEV_*l*_) > Th_*GEV*_
**Outputs:** Cluster{Cluster_1_,Cluster_2_,Cluster_3_,,Cluster_*k*_}

### Microstate Sequence Characteristics

After microstate classes are identified, the original individual EEG data can be labeled as a microstate sequence, with fitting back of these microstate classes to topographies at sample point. Temporal parameters can be extracted as features for further analysis and can also be compared between different experimental conditions or between groups of subjects.

#### Backfitting

Microstate classes are assigned to EEG at each time frame (or index of GFP peaks) considering the highest spatial correlation (see Eq. 5). The maximum spatial correlation determines the microstate label *L_t*. In the fitting process, temporal smoothing (Pascual-Marqui et al., 1995; Poulsen et al., 2018) is applied to avoid interruptions in spontaneous EEG sequences with a lot of unwanted noise—that is, class assignments are based on topographical similarity with microstate classes and the microstate labels of samples prior to and following the EEG sample. Different temporal parameters and statistical analyses will be performed after class assignments for every subject.

#### Temporal Parameters

EEG microstate sequences (EEG-MS) are symbolic time series related to potential neurophysiological relevance. The temporal dynamic characteristics of EEG-MS can be described by the following parameters. These statistical parameters mainly represent the activation strength, the spatial configuration, and the temporal attributes of microstates:

(1) Duration (ms): This refers to the average length of continuous sequences belonging to a given microstate class.
(2) Occurrence: This indicates the average frequency in which a microstate class is present per second. It is computed by taking the number of segments belonging to a microstate class divided by the whole analysis duration (in seconds).
(3) Time coverage (%): This represents the proportion of a specified microstate that is active during the whole analysis time.

GEV (%): This parameter is the percentage of explained variation of a given microstate class, described in Eq. 4.

#### Transition Probabilities

Transition probabilities can be derived to quantify the probabilities of a certain class switched to other classes. The transition probability between two states is given as *T*_*ij*_=*P*(*X*_*t* + 1_=*S*_*j*_|*X*_*t*_=*S*_*i*_). A Markov chain describes the probability distribution of the system either remaining in that state or transitioning to a different state for the next time point. In this study, separate transition probabilities are computed and compared for each of the four conditions (high vs. low valence and high vs. low arousal).

### Statistical Analysis

Statistical analyses were performed by using in-house scripts. Each microstate parameter was compared on the valence and arousal dimension separately. The trial is labeled as “high” group if its dimension value is higher than 4.5 and “low” group if its dimension value is lower than 4.5. To evaluate group differences in the microstate parameters mentioned above, we used Wilcoxon rank–sum statistic test for comparisons (Musaeus et al., 2019; Chu et al., 2020). The Wilcoxon rank–sum test is a nonparametric approach. It allows us to compare two populations where the underlying distributions are not normal but that do have similar shapes.

## Results

### Microstate Class Spatial Topographies

#### Microstate Classes

**FIGURE 4:**
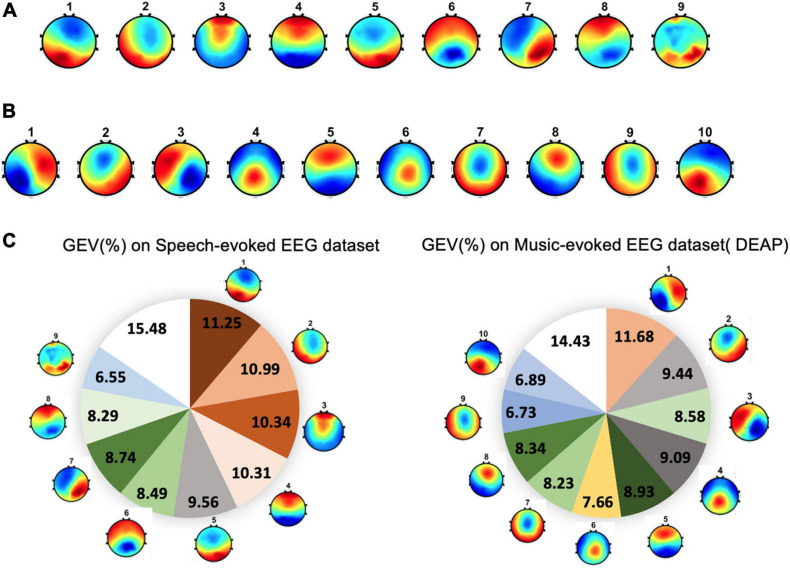
The topographical maps of the microstates across subjects. **(A)** Microstates from speech-evoked emotion cognitive experiment. **(B)** Microstates from music-evoked datasets. **(C)** The global explained variance (GEV) of each microstate for two datasets.

For dataset 1, the group-level clustering revealed nine optimal microstate classes for emotional speech-evoked EEG. These nine microstate topography templates are illustrated in Figure 4A. The topographies are labeled as #1–9. For dataset 2, the microstate analysis identified 10 microstates for emotional music video-evoked EEG (see Figure 4B).

#### Global Explained Variance

The performance of the microstate segmentation algorithm is reported in terms of the GEV, which estimates the portion of EEG point topography that can be explained by microstates. For dataset 1, the nine EEG microstate classes together explained around 85% of the data in global field power peaks. The GEV of each microstate class ranged from 6.55 to 11.25% (see Figure 4C). For dataset 2, ten microstates explained 86% of the variance of all global field power peaks. Correspondingly, the GEV of each microstate class fluctuates between 6.73 and 11.68%.

#### Global Map Dissimilarity

GMD is calculated as a measure of topographic differences of microstate maps. For dataset 1, the GMD matrix across different microstates is shown in Table 3. The GMD ranged from 0.10 to 0.25 (mean = 0.18, *SD* = 0.06). Table 4 presents the GMD between different microstates of dataset 2. The average GMD is 0.25 (*SD* = 0.08). The range of the GMD is 0.10–0.34.

**TABLE 3:** The global map dissimilarity (GMD) between different microstates of dataset 1.

GMD		Microstates from dataset 1								
		#1	#2	#3	#4	#5	#6	#7	#8	#9
	#1	0	0.11	0.23	0.25	0.12	0.23	0.15	0.23	0.10
**Microstates from dataset 1**										
	#2	0.11	0	0.23	0.23	0.14	0.21	0.17	0.21	0.11
	#3	0.23	0.23	0	0.11	0.24	0.11	0.23	0.12	0.23
	#4	0.25	0.23	0.11	0	0.25	0.11	0.22	0.10	0.24
	#5	0.12	0.14	0.24	0.25	0	0.23	0.10	0.24	0.10
	#6	0.23	0.21	0.11	0.11	0.22	0	0.23	0.10	0.23
	#7	0.15	0.17	0.23	0.22	0.10	0.23	0	0.24	0.10
	#8	0.23	0.21	0.12	0.10	0.24	0.10	0.24	0	0.23
	#9	0.10	0.11	0.23	0.24	0.10	0.23	0.10	0.23	0

**TABLE 4:** The GMD between different microstates of dataset 2 (Dataset for Emotion Analysis using Physiological signals, DEAP).

GMD		Microstates from DEAP									
		#1	#2	#3	#4	#5	#6	#7	#8	#9	#10
	#1	0	0.25	0.29	0.30	0.18	0.24	0.28	0.14	0.31	0.33
**Microstates from DEAP**											
	#2	0.25	0	0.33	0.19	0.34	0.22	0.11	0.33	0.16	0.16
	#3	0.29	0.33	0	0.30	0.14	0.32	0.28	0.20	0.23	0.28
	#4	0.30	0.19	0.30	0	0.33	0.11	0.24	0.31	0.25	0.11
	#5	0.18	0.34	0.14	0.33	0	0.30	0.32	0.10	0.30	0.34
	#6	0.24	0.22	0.32	0.11	0.30	0	0.29	0.26	0.31	0.17
	#7	0.28	0.11	0.28	0.24	0.32	0.29	0	0.33	0.11	0.19
	#8	0.14	0.33	0.20	0.31	0.10	0.26	0.33	0	0.33	0.34
	#9	0.31	0.16	0.23	0.25	0.30	0.31	0.11	0.33	0	0.194
	#10	0.33	0.16	0.28	0.11	0.34	0.17	0.19	0.34	0.19	0

### Temporal Parameters

It is controversial whether the first-order Markov model can capture the complex temporal dependencies for a longer time series of minutes (von Wegner et al., 2017). The duration of one trial in DEAP is 60 s. The duration is 5 s in the emotional speech-evoked cognitive experiment. Therefore, the microstate sequence characteristics are evaluated on the speech-evoked EEG dataset. We compared the temporal parameters of microstates in valence and arousal dimensions separately. We divided the trials into two groups based on the valence or arousal level. The trial is labeled as “high” group if its valence (or arousal) value is higher than 4.5 and as “low” group if it is lower than 4.5.

The comparison results are shown in Table 5. For the valence dimension, the mean duration, occurrence, time coverage, and GEV are investigated for the high valence and the low valence groups. The Wilcoxon rank–sum statistic test was used to identify statistically significant differences between high/low conditions for each microstate class in every temporal parameter. The significance level is set to 5%. The significant group differences are marked with an asterisk. The result revealed that the duration of microstate #3 is significantly increased during the response to a high valence stimulus (*p* = 0.02). No significant differences in occurrence, time coverage, and GEV between the groups are found.

**TABLE 5:** Means for all microstate parameters of speech-evoked EEG signals.

Microstate classes										
Temporal parameters		#1	#2	#3	#4	#5	#6	#7	#8	#9
		
		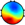	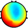	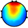	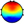	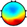	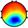	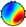	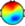	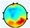
**Mean duration, ms (SD)**										
Valence	High	97.58 (17.4)	106.33 (21.0)	110.73 (22.5)	74.12 (15.6)	87.19 (19.3)	107.06 (23.9)	104.96 (20.8)	83.89 (16.8)	69.03 (28.1)
	Low	98.98 (19.6)	107.26 (19.5)	102.80 (21.8)	73.85 (17.5)	82.46 (17.0)	105.71 (22.5)	103.66 (19.5)	80.56 (18.6)	67.27 (227.5)
	*P*-value	0.53	0.22	0.02*	0.83	0.86	0.77	0.51	0.79	0.79
Arousal	High	98.32 (19.0)	106.77 (20.1)	105.21 (22.8)	73.82 (16.8)	83.18 (17.5)	106.64 (23.3)	103.86 (19.7)	81.60 (18.4)	67.66 (27.8)
	Low	102.72 (23.9)	110.36 (17.2)	98.90 (18.9)	75.19 (22.8)	89.18 (24.3)	98.55 (20.1)	105.45 (18.3)	78.48 (13.1)	68.32 (30.5)
	*P*-value	0.11	0.09	0.05*	0.64	0.52	0.05*	0.49	0.79	0.73
**Frequency of occurrence, counts/s (SD)**										
Valence	High	1.20 (0.6)	1.51 (0.6)	1.30 (0.6)	0.46 (0.3)	0.67 (0.4)	1.30 (0.6)	1.44 (0.5)	0.78 (0.5)	0.47 (0.2)
	Low	1.23 (0.6)	1.55 (0.5)	1.35 (0.7)	0.45 (0.3)	0.68 (0.4)	1.31 (0.6)	1.45 (0.5)	0.74 (0.5)	0.51 (0.2)
	*P*-value	0.52	0.50	0.49	0.93	0.86	0.56	0.84	0.64	0.27
Arousal	High	1.22 (0.6)	1.54 (0.6)	1.34 (0.7)	0.45 (0.3)	0.68 (0.4)	1.31 (0.6)	1.43 (0.5)	0.75 (0.5)	0.51 (0.2)
	Low	1.31 (0.6)	1.57 (0.5)	1.25 (0.6)	0.43 (0.3)	0.65 (0.3)	1.31 (0.8)	1.62 (0.6)	0.71 (0.4)	0.44 (0.2)
	*P*-value	0.20	0.62	0.39	0.55	0.72	0.76	0.04*	0.70	0.74
**Ratio of time coverage, % (SD)**										
Valence	High	12.11 (6.4)	16.57 (8.0)	15.84 (9.3)	4.13 (2.7)	6.48 (3.5)	14.29 (6.9)	15.53 (6.6)	7.26 (4.8)	7.74 (1.6)
	Low	12.79 (6.6)	17.02 (7.0)	15.22 (9.3)	4.02 (2.5)	6.37 (3.8)	14.59 (8.2)	15.16 (6.1)	7.12 (4.7)	7.67 (1.8)
	*P*-value	0.45	0.37	0.78	0.97	0.92	0.65	0.82	0.64	0.58
Arousal	High	12.52 (6.5)	16.85 (7.3)	15.51 (9.4)	4.06 (2.5)	6.36 (3.7)	14.61 (8.0)	15.10 (6.2)	7.21 (4.8)	7.73 (1.8)
	Low	14.02 (6.8)	17.64 (5.4)	13.59 (7.4)	3.88 (2.6)	6.88 (3.9)	13.27 (7.8)	17.13 (6.6)	6.49 (3.7)	7.07 (1.7)
	*P*-value	0.09	0.32	0.20	0.71	0.87	0.25	0.05*	0.58	0.68
**Global explained variance, % (SD)**										
Valence	High	6.35 (4.3)	6.38 (3.8)	9.22 (6.8)	3.65 (2.9)	3.82 (2.3)	6.01 (4.3)	4.87 (2.7)	4.84 (3.9)	3.06 (0.6)
	Low	6.53 (4.5)	6.79 (4.0)	9.08 (7.2)	3.27 (2.2)	3.64 (2.2)	6.16 (4.0)	4.82 (2.5)	4.58 (3.5)	2.90 (0.5)
	*P*-value	0.78	0.21	0.91	0.67	0.93	0.69	0.93	0.68	0.47
Arousal	High	6.42 (4.4)	6.62 (4.0)	9.21 (7.1)	3.38 (2.4)	3.65 (2.3)	6.20 (4.1)	4.76 (2.5)	4.71 (3.7)	2.97 (0.5)
	Low	7.37 (4.0)	7.59 (2.5)	7.97 (6.4)	3.19 (2.7)	4.13 (1.6)	5.16 (3.6)	5.65 (2.5)	3.78 (2.8)	2.67 (0.5)
	*P*-value	0.12	0.08	0.39	0.71	0.72	0.32	0.03*	0.54	0.92

*The asterisk indicates significant difference (*p* ≤ 0.05).*

For the arousal dimension, microstates #3 and #6 had a striking increase in duration for high arousal (*p* = 0.05). On the other hand, the occurrence, temporal coverage, and GEV of microstate #7 slumped during the same period for high arousal.

**FIGURE 5:**
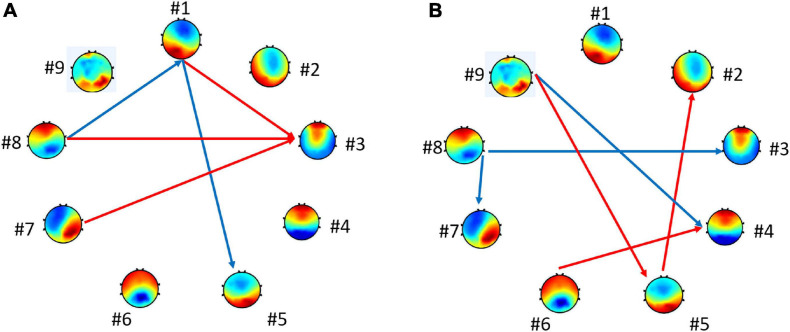
Connections with the statistically significant difference between groups. The blue arrows represent *p* < 0.05. The red arrows represent *p* < 0.10 for **(A)** high vs. low valence groups and for **(B)** high vs. low arousal groups.

Further tests examined the model of transition probabilities for valence and arousal, respectively. Table 6 depicted the statistically significant differences (*p*-value) of directions of transitions between high- vs. low-level groups. For valence, the statistical analysis unraveled the significant differences between high and low groups in five transitions: from microstate #1 to #3, #7 to #3, and #8 to #3 (*p* < 0.10) and from microstate #1 to #5 and #8 to #1 (*p* < 0.05). For arousal, six transitions have significant differences: from microstate #9 to #5, #5 to #2, and #6 to #4 (*p* < 0.10) and from #9 to #4, #8 to #3, and #8 to #7 (*p* < 0.05). Figure 5 highlights the directions of transitions that show significant differences.

**TABLE 6:** The differences (*p*-value) of transition probabilities between high and low valence or arousal.

→	Dimensions	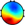	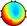	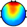	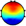	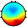	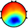	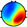	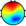	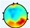
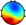	Valence	–	0.99	0.09	0.47	0.003	0.97	0.47	0.87	0.78
	Arousal	–	0.54	0.56	0.48	0.77	0.42	0.65	0.61	0.94
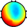	Valence	0.29	–	0.48	0.49	0.10	0.80	0.29	0.75	0.88
	Arousal	0.76	–	0.98	0.86	0.20	0.98	0.12	0.64	0.86
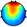	Valence	0.66	0.83	–	0.79	0.66	0.12	0.20	0.93	0.57
	Arousal	0.91	0.43	–	0.18	0.26	0.94	0.45	0.61	0.19
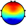	Valence	0.72	0.41	0.75	–	0.31	0.28	0.66	0.48	0.08
	Arousal	0.44	0.53	0.91	–	0.46	0.12	0.41	0.74	0.38
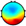	Valence	0.10	0.70	0.72	0.76	–	0.07	0.67	0.98	0.83
	Arousal	0.29	0.08	0.21	0.64	–	0.51	0.13	0.37	0.26
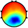	Valence	0.29	0.19	0.43	0.12	0.20	–	0.23	0.24	0.24
	Arousal	0.42	0.48	0.23	0.09	0.40	–	0.37	0.36	0.76
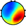	Valence	0.99	0.34	0.06	0.28	0.32	0.89	–	0.32	0.48
	Arousal	0.12	0.87	0.68	0.43	0.92	0.52	–	0.43	0.70
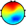	Valence	0.04	0.72	0.06	0.51	0.68	0.22	0.96	–	0.26
	Arousal	0.21	0.46	0.04	0.77	0.60	0.98	0.0002	–	0.71
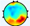	Valence	0.74	0.84	0.65	0.93	0.97	0.98	0.91	0.92	–
	Arousal	0.67	0.65	0.12	0.01	0.08	0.23	0.78	0.21	–

### Emotion Recognition Results

In order to verify the effectiveness of our feature sets, we firstly captured the EEG data from the public DEAP dataset to validate our framework. Then, the proposed feature extraction was applied to the speech-evoked EEG dataset.

A fivefold cross-validation method is adopted to evaluate the performance: the dataset is split into fivefolds. In each iteration, onefold is used to test the model, and the rests serve as the training set. The process is repeated until each fold has been used as the training set.

For the two-class classification problem, the accuracies are measured using

(7) $$\mathrm{Accuracy}=\frac{TP+TN}{TP+TN+FN+FP}$$

where TP, TN, FP, and FN denote true positive, true negative, false positive, and false negative, respectively.

#### Performance on DEAP Dataset

The dataset is separated into high–low classes by valence or arousal dimension. Each class is determined by the positivity of arousal and valence ratings. Valence and arousal levels higher than 4.5 are high and *vice versa*.

Considering temporal dependencies more complex than the first Markov models, von Wegner et al. (2017) suggested that the geometric distribution of microstate durations for short EEG time series was up to a duration of 16 s. In DEAP, the duration of EEG signals is 60 s. Therefore, we segment each signal using a moving window with a length of 5 s to evaluate short-time identifiability.

We perform three experiments on the microstate-related feature sets. We first use four temporal parameters (duration, occurrence, time coverage, and GEV) as features to obtain accuracies for the valence and arousal dimensions and later use transition probabilities as features to obtain the accuracies. Finally, we combine temporal parameters and transition probabilities as a feature set to measure performance. The extracted features are fed into the support vector machine (SVM) for classification. SVM is widely used for emotion recognition, which has promising properties in many fields. We also carry out comparisons of other features that exist in the works of literature.

The accuracy results of high/low valence and arousal are given in Table 7. The four temporal parameters with SVM yield accuracy rates of 72.5 and 72.1% for high/low valence and high/low arousal, while the transition probabilities have scores of 74.4 and 73.9%, respectively. The highest scores of 75.8% for valence and 77.1% for arousal are obtained by combining temporal parameters and transition probabilities. Our methods are compared to other states-of-the-art which use the DEAP dataset. According to the comparison table, our study has higher accuracy rates than the previous studies. The results demonstrate that the parameters derived from microstate sequences are promising features for characterizing the dynamics of neural activity and recognizing emotion from EEG signals.

**TABLE 7:** The classification accuracies of different feature sets on dataset 2 (Dataset for Emotion Analysis using Physiological signals, DEAP).

References	Feature set	Classifier	Accuracy	
			Valence (%)	Arousal (%)
Mert and Akan (2018)	MEMD, PSD, Entropy, Hjorth, IMF energy, energy ratios	k-NN ANN	67.0 72.7	51.0 75.0
Zhuang et al. (2017)	EMD, TSD, PD, NE	SVM	69.1	71.9
Daimi and Saha (2014)	DT-CWPT	SVM	65.3	66.9
	Temporal parameters	SVM	72.5	72.1
	Transition probabilities	SVM	74.4	73.9
This study	Temporal parameters +			
	transition probabilities	SVM	**75.8**	**77.1**

*DT-CWPT, dual-tree complex wavelet packet transform time–frequency features; PSD, power spectral density; EMD, empirical mode decomposition.*

#### Performance on Speech-Evoked EEG Signals

In this section, the performances of microstate characteristic features are evaluated on the emotional speech-evoked EEG dataset.

Three different classifiers are applied to three feature sets—that is, SVM, random forest, and artificial neural network (ANN). From Table 8, there is no significant difference among the three classifiers. The performance of the features extracted in this research is not affected by the type of classifiers. The highest accuracy is obtained by combining temporal parameters and transition probabilities as the feature set for valence and arousal. In valence recognition, the highest accuracy is 74.2% with the SVM classifier. For arousal, it is 72.3% with ANN.

**TABLE 8:** The classification accuracies of different feature sets on speech-evoked EEG signals.

Dataset	Feature set	Classifier	Accuracy	
			Valence (%)	Arousal (%)
	Temporal	Support vector machine (SVM)	71.8	68.8
	parameters	Random forest (RF)	72.0	67.9
		Artificial neural network (ANN)	72.3	69.5
		SVM	69.9	70.5
This study	Transition probabilities	RF	68.5	68.3
		ANN	70.4	69.8
	Temporal parameters + transition probabilities	SVM	**74.2**	71.9
		RF	73.1	70.7
		ANN	73.9	**72.3**

## Discussion

In this study, we applied the microstate analysis to the emotional auditory response. Our proposed method DTAAHC revealed that nine template maps best described the entire dataset, explaining ∼85% of the global variance for speech-evoked EEG. For music-evoked EEG, 10 template maps explain ∼86% of the data. In previous visual research, Gianotti et al. (2008) studied the temporal dynamics of the neural activity that responded to emotional words and picture stimulus using ERP microstate analysis. In the emotional word experiment, 11 sequential microstates were identified. Among the 11 microstates, four of them were valence-sensitive and two of them were arousal-sensitive. In the emotional picture experiment, the microstate analysis identified 15 sequential microstates. Five of the fifteen and two of the fifteen microstates were valence-sensitive and arousal-sensitive, respectively. Although four prototypical microstate classes were useful to compare or complement results across different studies, several studies also suggested that the number of microstate classes was explicitly driven by the data. Muthukrishnan et al. (2016) performed the microstate analysis in a visuospatial working memory task. The optimal number of clusters was determined by the cross-validation criterion without prior assumptions. D’Croz-Baron et al. (2019) investigated that six template microstate maps can best describe the dataset across the autism spectrum disorder and neurotypical controls. In research of schizophrenia (Soni et al., 2018, 2019), four to six microstate maps were clustered, which related to the conditions of the experiments. Michel and Koenig (2018) discussed a meta-criterion for the optimal number of clusters. They suggested that the most appropriate choice was a pragmatic compromise between the needs for specificity and generalizability.

The four prototypical microstates exhibited highly similar topographies across studies and were consistently labeled as class A, B, C, and D. Microstate A exhibits a left–right orientation, map B exhibits a right–left orientation, map C exhibits an anterior–posterior orientation, and map D exhibits a fronto-central maximum (Michel and Koenig, 2018). In terms of the orientation of the electrical axis, we relate some microstates of our study to four prototypical microstates. Here we mark maxima as “+” and minima as “-.”In our emotional speech-evoked cognitive experiment, three microstates (#3, #4, and #8) are characterized by fronto-central orientation of the maxima which are similar to map D (Santarnecchi et al., 2017; da Cruz et al., 2020). Some studies suggest that microstate D is associated with attention network activity (Britz et al., 2010; Milz et al., 2016). For the music-evoked EEG dataset, microstates #5 and #8 exhibit fronto-central maximum.

In the speech-evoked emotion experiment, microstates #1, #2, and #5 have an anterior(-)-posterior(+) orientation which is consistent with map C(Santarnecchi et al., 2017; Seitzman et al., 2017; Al Zoubi et al., 2019). Microstate #6 has an anterior(+)-posterior(−) orientation which is consistent with map C in some studies (Hernandez et al., 2016; Pipinis et al., 2017; da Cruz et al., 2020). In the music-evoked cognitive experiment, microstates #2 and #10 have an anterior(−)-posterior(+) orientation which is somewhat alike to map C.

In the speech-evoked emotion experiment, microstate #7 shows a left anterior (−)–right posterior (+) location of the extrema. It is similar to map B (Khanna et al., 2014; Santarnecchi et al., 2017). In the music-evoked cognitive experiment, microstate #3 has a left anterior(+)–right posterior(-) orientation which is alike to map B in some studies (Milz et al., 2017; Pipinis et al., 2017; da Cruz et al., 2020).

In the music-evoked cognitive experiment, microstate #1 has a left posterior(−)–right anterior(+) orientation which is consistent with map A in the studies (Tomescu et al., 2015; Pipinis et al., 2017; da Cruz et al., 2020).

We also identify some microstates which have significant differences with prototypical microstates. In the speech-evoked emotion experiment, microstate #9 has a local extremum in posterior (+). In the music-evoked emotion experiment, microstates #4 and #6 exhibit local maxima in posterior. Microstates #7 and #9 show local minima at the axis center.

For future research, the relationship between microstates and brain functions can be explored using source localization. Some computational approaches, e.g., distributed linear inverse solution (LAURA) (de Peralta Menendez et al., 2004), can help understand the brain source activation in terms of intracranial generators.

We further delved into the temporal characteristics of microstates for emotional speech perception. The Wilcoxon rank–sum test was used to analyze the statistical differences of the microstate parameters between different groups. For the valence dimension, the results indicated that the mean duration of microstate #3 (active prefrontal cortex) in the high group was longer than that in the low group. For arousal dimension, three microstates had significant differences between high and low group. Specifically, the mean duration of microstates #3 and #6 (active frontal lobe) in the high group was longer than those in the low group. The occurrence, coverage, and GEV of microstate #7 (active temporal lobe) had significant differences between the high and low groups. In previous research, Gianotti et al. (2008) found that five of the 15 microstates were different for pleasant vs. unpleasant pictures, and two of the 15 microstates were different for high- vs. low-arousing pictures. However, it was difficult to compare this work with our study directly since visual and auditory information activated different cortices.

## Conclusion

The main purpose of this study is to extract novel features based on EEG microstates for emotion recognition. Determining the optimal number of microstates automatically is a challenge for applying microstate analysis to emotion. To overcome the limitation, this research proposed DTAAHC. The proposed method identified 10 microstates on a public music-evoked EEG dataset (DEAP) and nine microstates on our recorded emotional speech-evoked EEG dataset. Subsequently, the microstate sequence characteristics were compared in the aspect of high/low valence or arousal conditions. Finally, these characteristics were fed into the classifier for emotion recognition. All the findings in this work suggested that the microstate sequence characteristics can effectively improve the performance of emotion recognition from EEG signals. We hope this work will stimulate future research to propose novel algorithms to reduce the limitation of microstate analysis and uncover more interesting mechanisms of the affective process, e.g., linking the source localization of microstates to brain functions can help understand the functional significance of these states.

## Data Availability Statement

The raw data supporting the conclusions of this article will be made available by the authors, without undue reservation.

## Ethics Statement

The studies involving human participants were reviewed and approved by the Heilongjiang Provincial Hospital. The patients/participants provided their written informed consent to participate in this study.

## Author Contributions

JC was involved in the conduct of the experiment, data analysis, and writing of the manuscript. HL, LM, and FS were involved in the conception, supervision, and manuscript review. HB was involved in the study design and conduct of the experiment. YS was involved in the study design and subject recruitment. All authors contributed to the article and approved the submitted version.

## Conflict of Interest

FS was employed by company Microsoft Research Asia. The remaining authors declare that the research was conducted in the absence of any commercial or financial relationships that could be construed as a potential conflict of interest.
