# Supplementary material for: Dual-Threshold-Based Microstate Analysis on Characterizing Temporal Dynamics of Affective Process and Emotion Recognition From EEG Signals
Source: Front Neurosci. 2021 Jul 14;15:689791. doi: 10.3389/fnins.2021.689791 (PMC8318040; doi:10.3389/fnins.2021.689791)
Supplement: Supplementary file 1 [file Table_1.DOCX]

Table 1. Description of the film clips

| Number | Film title | Target emotion(pair) | | | | | |
| --- | --- | --- | --- | --- | --- | --- | --- |
|  |  | Anger | Disgust | Fear | Joy | Sadness | Surprise |
| 1 | Absolutely Anything | 1 |  | 1 |  |  |  |
| 2 | August Rush | 1 | 1 |  | 1 | 1 | 1 |
| 3 | Ant-man | 1 |  |  | 1 |  |  |
| 4 | Alice |  |  |  |  |  | 1 |
| 5 | Breakfast at Tiffany's | 2 |  |  |  |  |  |
| 6 | Con Air | 1 |  |  |  |  | 1 |
| 7 | Doctor Strange |  |  |  |  |  | 1 |
| 8 | Death on the Nile |  |  |  | 1 |  |  |
| 9 | Edge of Tomorrow |  |  |  |  |  | 2 |
| 10 | Finding Nemo |  |  | 4 |  | 1 | 3 |
| 11 | Forrest Gump |  | 1 |  |  | 1 |  |
| 12 | Flipped | 1 |  |  | 1 | 1 |  |
| 13 | Frequency |  |  |  |  |  | 1 |
| 14 | Freedom Writers |  |  |  |  | 1 |  |
| 15 | Hoodwinked | 1 |  |  |  |  |  |
| 16 | Hitch |  |  |  | 2 | 1 | 1 |
| 17 | Inside Out | 1 | 1 | 1 | 4 | 2 | 2 |
| 18 | Inside Man |  |  | 2 |  |  |  |
| 19 | Man of Steel | 1 | 1 |  |  |  |  |
| 20 | Moana | 1 |  |  | 1 | 2 | 1 |
| 21 | Maze |  |  | 1 |  |  |  |
| 22 | Pixels |  |  |  |  | 1 |  |
| 23 | Penguins of Madagascar | 1 | 1 |  | 1 |  |  |
| 24 | Pride And Prejudice | 2 | 1 |  | 2 | 1 | 1 |
| 25 | Romeo and Julie |  |  |  |  | 2 |  |
| 26 | Revolution road | 2 |  |  | 1 |  |  |
| 27 | Seven |  | 1 | 2 |  |  |  |
| 28 | The Spiderwick Chronicles |  | 1 |  |  |  | 1 |
| 29 | The Croods |  | 1 |  |  |  |  |
| 30 | The Devil Wears Prada |  | 2 |  |  |  |  |
| 31 | The Holiday |  | 1 |  |  | 1 | 1 |
| 32 | The Lake House |  | 1 |  | 1 | 2 |  |
| 33 | The Prestige | 1 |  |  |  |  |  |
| 34 | The Silence of the Lambs |  |  | 4 |  |  |  |
| 35 | Thelma & Louise |  |  | 1 | 1 |  | 1 |
| 36 | The BFG |  | 3 | 1 | 2 |  |  |
| 37 | Titanic | 2 | 4 | 1 |  |  |  |
| 38 | Toy Story |  |  |  |  |  | 2 |
| 39 | Vertical Limit |  |  |  |  | 1 |  |
| 40 | Zootopia | 1 |  | 2 | 1 | 2 |  |
